# Power calculation for mosquito bioassays: Quantifying variability in the WHO tube bioassay and developing sample size guidance for the PBO synergism assay using a Shiny application

**DOI:** 10.12688/gatesopenres.16123.1

**Published:** 2024-09-12

**Authors:** Frank Mechan, Giorgio Praulins, Jack Gillespie, Katherine Gleave, Annabel Murphy-Fegan, Daniel P. McDermott, David Weetman, Rosemary Susan Lees

**Affiliations:** 1 Innovation To Impact (I2I), Department of Vector Biology, Liverpool, Liverpool, L35QA, UK; 2 Vector Biology, Liverpool School of Tropical Medicine, Liverpool, England, UK

**Keywords:** Malaria, vector control, bioassay, mosquito, insecticide resistance, resistance monitoring, variability, sample size, power analysis

## Abstract

**Background:**

The WHO tube bioassay is a method for exposing mosquitos to determine susceptibility to insecticides, with mortality to discriminating doses <98% indicating possible resistance and <90% confirming resistance. This bioassay is also used for synergism testing to assess if susceptibility is restored by pre-exposure to the synergist piperonyl butoxide.

**Methods:**

Here we perform testing with pyrethroid-susceptible and pyrethroid-resistant *An. gambiae* to quantify the variability of the WHO tube bioassay and identify its sources. These estimates of within and between day variability are then used to evaluate the power of the bioassay to detect a mortality difference between pyrethroid-only and pyrethroid-PBO.

**Results:**

We show that approximately two-thirds of variation occurs between days, with the pyrethroid-susceptible strain twice as variable as the pyrethroid-resistant strain. The total number of mosquitoes in the tube and their bodyweight contributes to approximately 10% of this variability. Changes in temperature and humidity, within a climate-controlled insectary, didn’t impact mortality. Using a simulation-based framework, we show that the current synergism guidelines, using a 4x4 design, can reliably detect a difference between 90% and 100% mortality (>90% power). However, as the mortality of either group gets closer to 50%, a 10% difference between groups is more difficult to reliably detect. In the worst-case scenario where the mortality of either group is 50%, the mortality difference must be >22.5% to be detected with 80% power. We provide an R shiny application to assess power for other comparisons.

**Conclusions:**

Our findings indicate that detecting synergism with the WHO tube assay is more difficult than assumed by the current WHO guidelines. Additionally, we demonstrate the value of using a Shiny application to make the outputs of simulation-based power analysis readily available to end-users, allowing them to determine the number of tubes needed to detect a given mortality difference.

## Introduction

The use of insecticides is a core component of global malaria control strategy, limiting contact between mosquito vectors and human populations ( Bhatt *et al.*, 2015; Wilson *et al.*, 2020). The most common strategies target the adult stages of the vector, primarily insecticide treated nets (ITNs) to prevent nighttime biting, sometimes supplemented by indoor residual spraying (IRS) to kill mosquitoes that rest inside dwellings ( WHO, 2023) ( Killeen, 2020). However, the continued efficacy of pyrethroid insecticides in malaria vector control is threatened by the widespread emergence of resistance in mosquito populations ( Hemingway *et al.*, 2016; Ranson & Lissenden, 2016). The long-term use of pyrethroid class insecticides throughout sub-Saharan Africa in both vector control and agriculture has resulted in a selective pressure in *Anopheles* mosquito populations towards adaptations that provide a protective effect against these tools ( Hancock *et al.*, 2020; Moyes *et al.*, 2020).

To manage insecticide resistance, it is necessary to implement detailed, routine resistance monitoring programmes on vector populations. The World Health Organization (WHO) provides guidance to national control programmes on identifying insecticide resistance in mosquito populations ( WHO, 2022a). The data generated through monitoring of resistance in vector populations allows control programmes to be adjusted appropriately, maximising the efficacy and longevity of interventions ( Kouassi *et al.*, 2023; Moyes *et al.*, 2020; WHO, 2022a). The WHO tube bioassay is a straightforward method for identifying evidence of insecticide resistance in a population, with 25 adult mosquitoes exposed to papers treated with insecticide for one hour. A mosquito population is considered resistant to an insecticide if mortality is less than 90% 24 hours post-exposure (with 90–98% mortality considered an indicator of possible resistance) ( WHO, 2022b). Increasingly importantly, the WHO tube assay can be used to evaluate the ability of a synergist to restore susceptibility to a pyrethroid-resistant mosquito population. If mosquitoes pre-exposed to the synergist piperonyl butoxide (PBO) subsequently exhibit increased mortality following exposure to a discriminating concentration of a pyrethroid insecticide, relative to those exposed to the pyrethroid alone, then this is evidence that resistance results from upregulation of P450 metabolic enzymes ( Edi *et al.*, 2014; Moyes *et al.*, 2021). 

The results of synergism testing are used to guide decision making when choosing to deploy ITNs containing PBO as part of a vector control programme ( WHO, 2023). It is therefore important that the threshold at which a mortality increase following PBO pre-exposure represents evidence of synergism and therefore metabolic resistance should be supported by robust statistical evidence, which will be dependent on sample size. A PBO assay should be designed so that an indication of synergism can be reliably distinguished from random fluctuations in mortality due to the variability inherent to bioassays ( Praulins *et al.*, 2022). Currently, WHO guidance indicates that with a sample size of four tubes of each treatment (a so-called ‘4x4’ design), this synergism threshold is a 10% difference in 24hr mortality between pyrethroid-only and pyrethroid-PBO treatments ( WHO, 2022c).

Where a sample size is fixed, identifying the effect size (mortality difference) that can be reliably detected by a statistical analysis is entirely dependent on the variability of the outcome. In the context of a WHO resistance assay with a fixed ‘4x4’ sample size, identifying the mortality difference that can be reliably detected requires quantification of the variability in mortality measured between tubes of the same treatment. Known sources of variation in a mosquito bioassay include genetic differences between mosquito populations, environmental differences (e.g. temperature, humidity, time of day), the number of mosquitoes per test item (tube in this case), and experimental technique (e.g., reagents, user variability/experience, measurement tools). Beyond the variability explained by known parameters is a level of unexplained variation (known as ‘noise’). While all variation is attributable to some cause, there are limits to what can be controlled or measured in a mosquito bioassay. Nonetheless, procedures to standardise these definable sources of variation and thus reduce the variability of the outcome make it possible to detect a smaller effect size than would otherwise be possible with the same sample size. The variability remaining after standardising procedures (the ‘noise’) can be used to calculate the minimum mortality difference between treatments that can be detected with the experimental design.

The purpose of this study was to identify and quantify as far as possible the sources of variation in WHO tube bioassays and use an estimate of the remaining level of ‘noise’ to determine appropriate sample size guidance for detecting a difference in 24hr mortality between pyrethroid-only and pyrethroid-PBO exposure treatments. This will help generate more robust data on which to determine the presence or absence of synergism and thus metabolic resistance mechanisms in a mosquito population and better inform decisions about where to distribute pyrethroid-PBO ITNs.

## Methods

### Mosquito rearing

Mosquito colonies used in this study were reared and maintained at the Liverpool Insect Testing Establishment (LITE) at the Liverpool School of Tropical Medicine (LSTM) as per the methods of ( Williams *et al.*, 2019). Mosquitoes were reared in environmentally controlled insectaries set at 26 ± 2 °C and 80 ± 5% relative humidity (RH), with a 12-hour light:dark cycle. Larvae were reared in purified water and fed on ground TetraMin® tropical flakes (Blacksburg, VA, USA). All mosquitoes tested were 2–5-day old adults and were always provided with ad libitum access to a 10% sucrose solution except during the tube exposure period. 

Two different *An. gambiae* strains were used in this study, pyrethroid-susceptible ‘Kisumu’ and pyrethroid resistant Tiassalé 13. These strains have been characterised previously by ( Williams *et al.*, 2019)

### WHO tube bioassays

The methods used here are taken from WHO guidance on testing insecticide susceptibility of adult in WHO tube tests ( WHO, 2022c), except where explicitly stated otherwise. Are mosquitoes tested were *An. gambiae s.s.* females 3–5 days old.

All bioassays were performed in environmentally controlled testing rooms (26 ± 2 °C and 80 ± 5% RH). Additionally, based on recent work by Odufuwa *et al.*, 2024 indicating that susceptibility varies across time of day, all assays were performed from 2–3pm each day. The insecticidal papers used were all treated with permethrin, with silicone oil used as a carrier, at LSTM using WHO guidelines ( WHO, 2022d). A different concentration of permethrin was used for the susceptible and resistant strains. The permethrin concentration chosen for the susceptible Kisumu strain was 0.03% (Sigma-Aldrich®, Merck catalogue no. 45614), based on a preliminary dose response assay which aimed to identify a concentration that resulted in approximately 50% mortality (median lethal concentration, LC _50, _was calculated using a probit analysis in PoloPlus, Version 2.1, LeOra Software, https://leora-software.com/). The permethrin concentration for the pyrethroid-resistant Tiassalé 13 strain was the WHO discriminating concentration of 0.05% ( WHO, 2022e).

While the current guidance for WHO tube bioassays ( WHO, 2022d) outlines that each tube should contain 25 mosquitoes (or as close to 25 as possible), for these experiments we allowed number of mosquitoes per tube to vary incidentally from 20–35 in order to investigate the impact of number per tube on the outcome. Exposures to the insecticidal papers lasted one hour. Each tube was scored for knockdown at 60 minutes and mortality at 24 hours post-exposure, though only 24hr mortality will be assessed here.

### Quantifying mosquito weight and size

A sample of approximately 25 mosquitoes were collected, from the same cohort of mosquitoes (testing cages) used to collect the mosquitoes that were exposed in the bioassays, in order to estimate body size. Three different metrics were used to quantify body size: wet weight, dry weight, and wing length.

To determine these metrics, mosquitoes were first incapacitated by placing them in a freezer for 30 minutes. To determine wet weight, each sample was placed on a Sartorius® balance Model TE214S (Göttingen, Germany) and this total weight divided by the number of mosquitoes. To obtain the dry weight of this sample, the mosquitoes were desiccated by storing them in a 25ml falcon tube with silica gel for at least one week. The dry weight was then measured and calculated in the same way as wet weight. To measure wing length, one wing from each mosquito was dissected and examined under a GXMMZs0745 microscope (GT Vision®, UK). Images were captured using a GXCAM™ Eclipse Camera (GT Vision, UK) and GXCAM™ software (Version 6.7, GT Vision, UK) and ImageJ™ (Version 1.54d, NIH, USA) used to measure the mosquito wing lengths.

### Statistical analysis

All analysis was performed in R studio™ (Posit PBC, MA, USA, https://posit.co/downloads/). Associations between parameters of interest and outcome (24-hour mortality) were assessed using Generalised Linear Mixed Models (GLMMs) with a binomial link function, performed using the ‘lme4’ package in R ( https://www.r-project.org/). The explanatory variables assessed in the GLMM were wet weight, dry weight, wing length, temperature, and humidity (conditions measured both at the beginning and end of the exposure period). The statistical significance of each parameter being assessed was evaluated using log likelihood ratio tests, where the explanatory power of a model with the parameter included was compared to an equivalent one without. To quantify random variation between observations, a random effect term was included for both individual tubes within a testing day and for date, this allowed ‘within-day’ and ‘between-day’ variance estimates to be extracted from the random effect component of the GLMM. The explanatory power of different models was compared using conditional R ^2^, combing both fixed and random effects ( Nakagawa & Schielzeth, 2013).

The coefficient of variation (as measure of the precision of the assays) was calculated by dividing the standard deviation of each days assays by the mean.

### Simulation study

Simulation-based power analysis is a process for establishing a suitable sample size for an experiment by simulating a very large number of datasets that could plausibly be collected (with the limits of these parameters based on the variance observed in the experimental component described above), then analysing the results to see how often an effect could be detected. Here, the aim was to determine what sample size (of tubes) was sufficient to reliably distinguish between two treatment groups (pyrethroid-only vs pyrethroid-PBO) in WHO tube synergism bioassays. The sample size needed for sufficient power (an 80% chance of detecting a difference, per convention) is dependent on the effect size (how much the difference is between treatments) and the variance between observations.

The simulation of realistic datasets is dependent on plausible assumptions about variance. Here, this was achieved by analysing the data collected in this study, extracting within-day and between-day variance from the laboratory testing as outlined above. Unique combinations of effect size (difference in mean mortality), within-day variance, and between-day variance were assessed across a range of sample sizes ( Table 1).

**Table 1.** Parameters assessed in power simulation.

Parameter variable	Possible values
Mortality difference	0 to 50% in 2.5% increments
Total number of tubes of each treatment	4,5,6,7,8,9,10
Number of testing days	1,2,3,4,5
Within-day variance	0.15, 0,20, 0.25, 0.30, 0.35
Between-day variance	0.40, 0.50, 0.60, 0.70, 0.80

In this study, 10,000 datasets were simulated for each combination of parameter values (effect size, sample size, and variance). For each set of parameter values, all datasets were analysed with a binomial GLM with a single fixed effect for treatment group. No random effects were included; testing day could in principle be included as a random effect however in these simulations the maximum number of day groups was five, making it inappropriate as a random effect ( Johnson *et al.*, 2015). A p value was extracted from the analysis using log-likelihood ratio tests (LRTs) (‘lmtest’ package in R, https://cran.r-project.org/web/packages/lmtest/index.html. The power reported for a specific parameter combination was the proportion of the 10,000 analyses where p<0.05. By comparing a range of sample sizes, we can identify the minimum number of tubes of each treatment group needed to affect a given effect size (e.g. a 10% difference in mortality).

## Results

### Variability in WHO tube bioassay results

**Figure 1.**
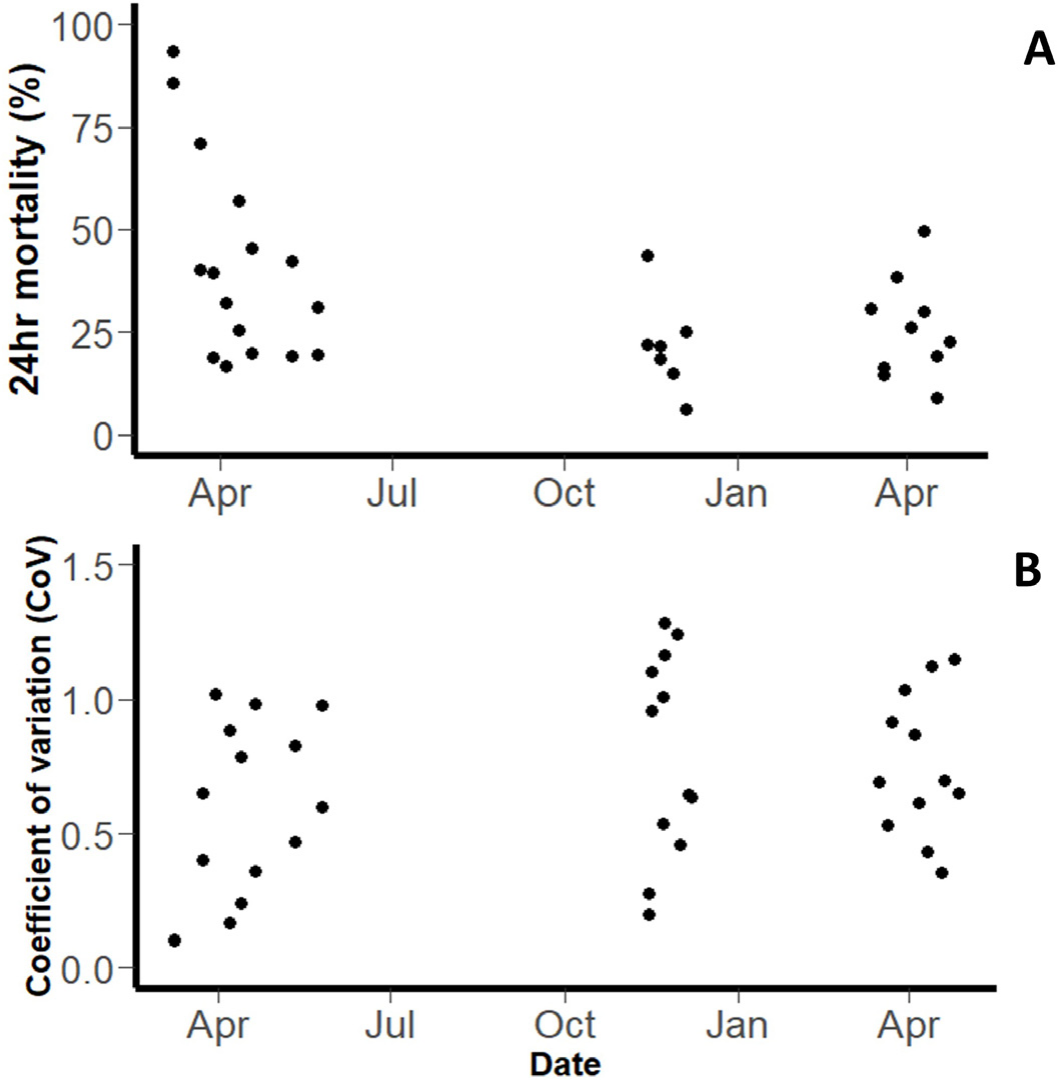
Outcomes of WHO tube assays with pyrethroid-susceptible *An. gambiae* Kisumu strain. Plot shows ( **A**) Mean 24hr mortality on each testing day and ( **B**) Coefficient of Variation. Each point represents the mean of all bioassays performed that day.

***Pyrethroid-susceptible strain (‘Kisumu’).*** A total of 10,258 mosquitoes were exposed in WHO tube bioassays, across a total of 404 tubes (across a total of 29 testing days). Overall, the mean 24hr mortality was 49.18% (95% CI: 37.19-65.04, Figure 1A). However, mortality was variable between days. The intra-assay precision (a measure of how consistent testing was on a given day) varied across the experimental period. The coefficient of variance (CoV) (a measure of inter-assay precision) for the WHO Tube bioassay was 0.719 overall ( Figure 1B).

The variation between tube tests performed on different days was much greater than the variation between tube tests on the same day. The within day standard deviation was 0.350 (equivalent to a variance of 0.123), and the between day standard deviation was 1.236 (equivalent to a variance of 1.529).

**Figure 2.**
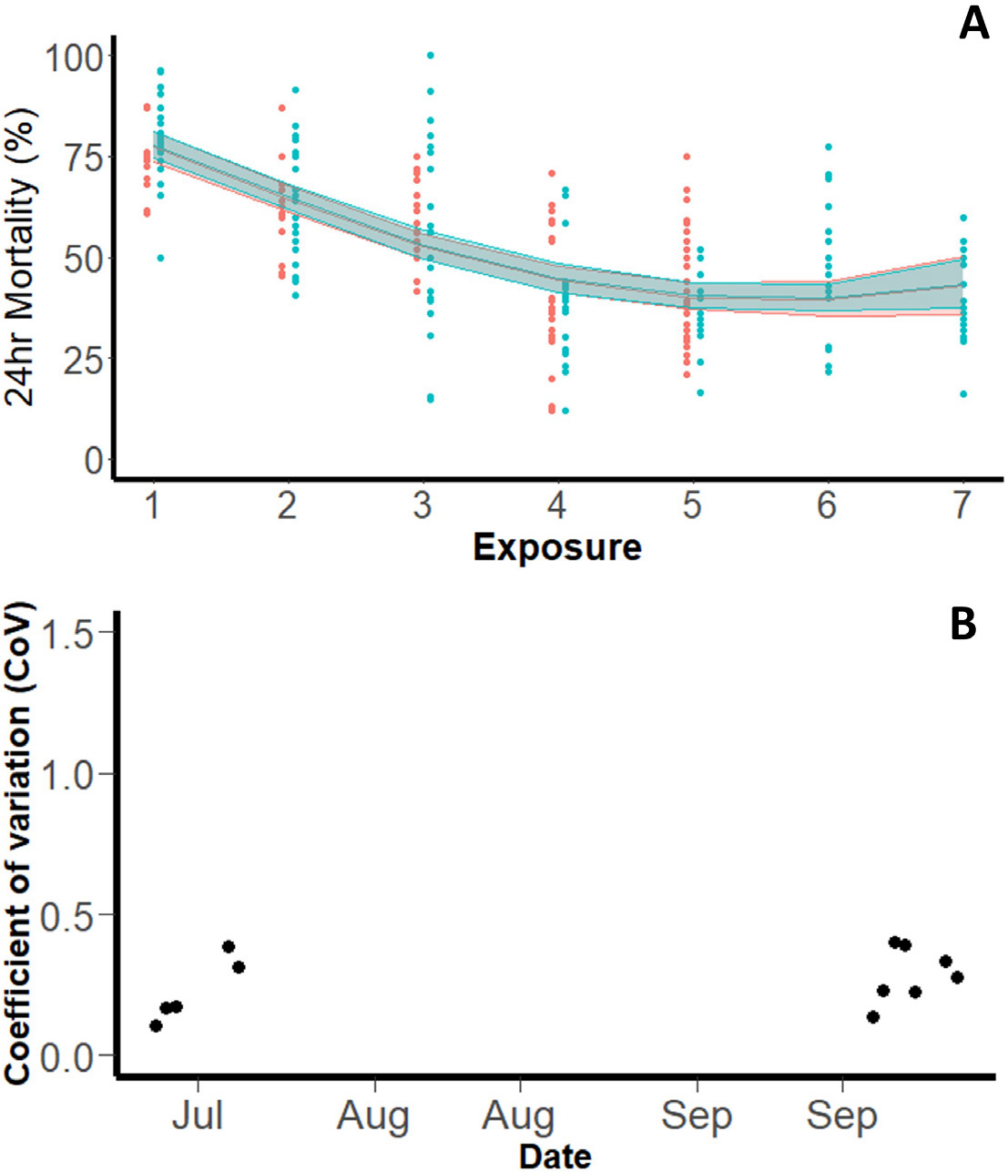
Outcomes of WHO tube assays with pyrethroid-resistant *An. gambiae* Tiassalé 13 strain. Panel ( **A**) shows mean 24hr mortality by day, the solid line represents the trend with 95% confidence interval (blue: July, red: September repeat). Panel ( **B**) shows Coefficient of Variation (Intra-assay precision) on each testing day.

***Pyrethroid-resistant (Tiassalé 13).*** A total of 6,332 pyrethroid-resistant Tiassalé 13 mosquitoes were exposed in WHO tube bioassays across 261 tubes (across a total of 12 testing days). Overall, the mean mortality with 0.05% permethrin, following PBO exposure, was 53.1% (95% CI: 44.44-61.72). The WHO protocol states that the same insecticide-treated papers can be used a maximum of six times, which is six cohorts of mosquitoes can be exposed to the same papers before new papers should be used ( WHO, 2022f). There was evidence from the testing done with the resistant Tiassalé 13 strain to suggest that 24hr mortality reduced with number of times a paper was used (tffhis was not assessed for Kisumu). On the first exposure, 24hr mortality was 77.88% (95% CI: 74.51-81.01) yet fell to 44.82% (95% CI: 41.76-48.76) on the fourth exposure (p<0.001) of mosquitoes to the same papers. There was no subsequent change in mortality after the fourth exposure. To confirm this observation, the experiment was repeated in full with the repeat extending the total number of times a paper was used to seven ( Figure 2A) (the only other difference being that papers were stored in the testing room between replicates in the first and in the fridge in the second). The same trend was observed, with the trendline statistically indistinguishable between the two repeats (p=0.4937).

The intra-assay precision for pyrethroid-resistant mosquitoes was 0.262 ( Figure 2B), substantively smaller than observed for pyrethroid-susceptible Kisumu mosquitoes. The variation between tubes on different days was overall greater than the variation between tubes on the same day, as with Kisumu. The within day standard deviation was 0.223 (equivalent to a variance of 0.049), and the between day standard deviation was 0.59 (equivalent to a variance of 0.349).

### Sources of variation in the WHO tube bioassay

To better understand the possible sources of inter- and intra-day variation (‘noise’), further analysis was performed on the data from these experiments to explore the impact of changes in different variables on the 24hr mortality of Kisumu in the WHO tube bioassay.

Assessment of the deviance explained by the variables included in the analysis indicated that the total number of mosquitoes in a tube and the total dry weight of the sample of mosquitoes from the testing cohort were the variables that had the greatest impact on mortality, contributing 5.90% and 6.27% of variance, respectively (or 10.19% when combined in the same model, slightly lower than the individual values as they are correlated due to total number and total weight being related).

**Figure 3.**
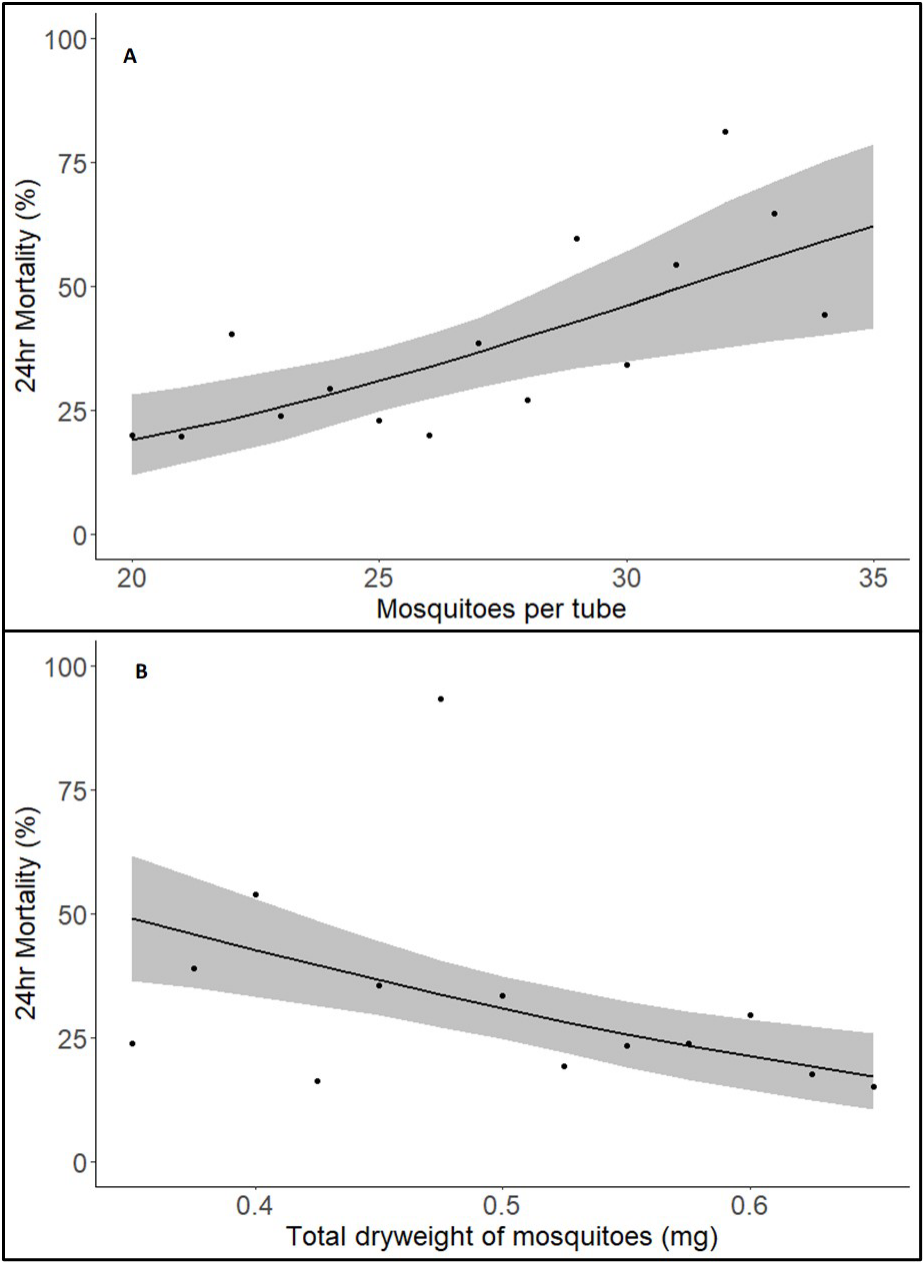
Relationship between 24hr mortality with Kisumu. ( **A**) mosquitoes per tube and ( **B**) dry weight of mosquito samples from same cohort. Solid line indicates predicted 24hr mortality with shaded area representing 95% CI. Each point represents the raw mean mortality of the data for that value on the x-axis.

The best fit model indicated that both mosquito number (p<0.001) and total dry weight (p<0.001) were predictors of mortality. For the number of mosquitoes in the tube, when all other variables are held constant, the probability of mortality increased by 2.84% for each additional mosquito (within the range 20–35) ( Figure 3A).

For the total dry weight of all mosquitoes in the tube, when all other factors were held constant, a 1SD increase above the mean (a 14% increase in total dryweight from 0.489mg to 0.557mg) resulted in a 7.91% decrease in mortality ( Figure 3B).

The mean wing length of mosquitoes in a tube did not predict 24hr mortality (p=0.18) and neither did the total wet weight of mosquitoes from each tube (p=0.372).

The impact of environmental variables on 24hr mortality was evaluated however neither temperature (p=0.086) nor relative humidity (p=0.408), as measured at the start of the exposure period, impacted the outcome.

### Implications of variability for detecting PBO synergism

**Figure 4.**
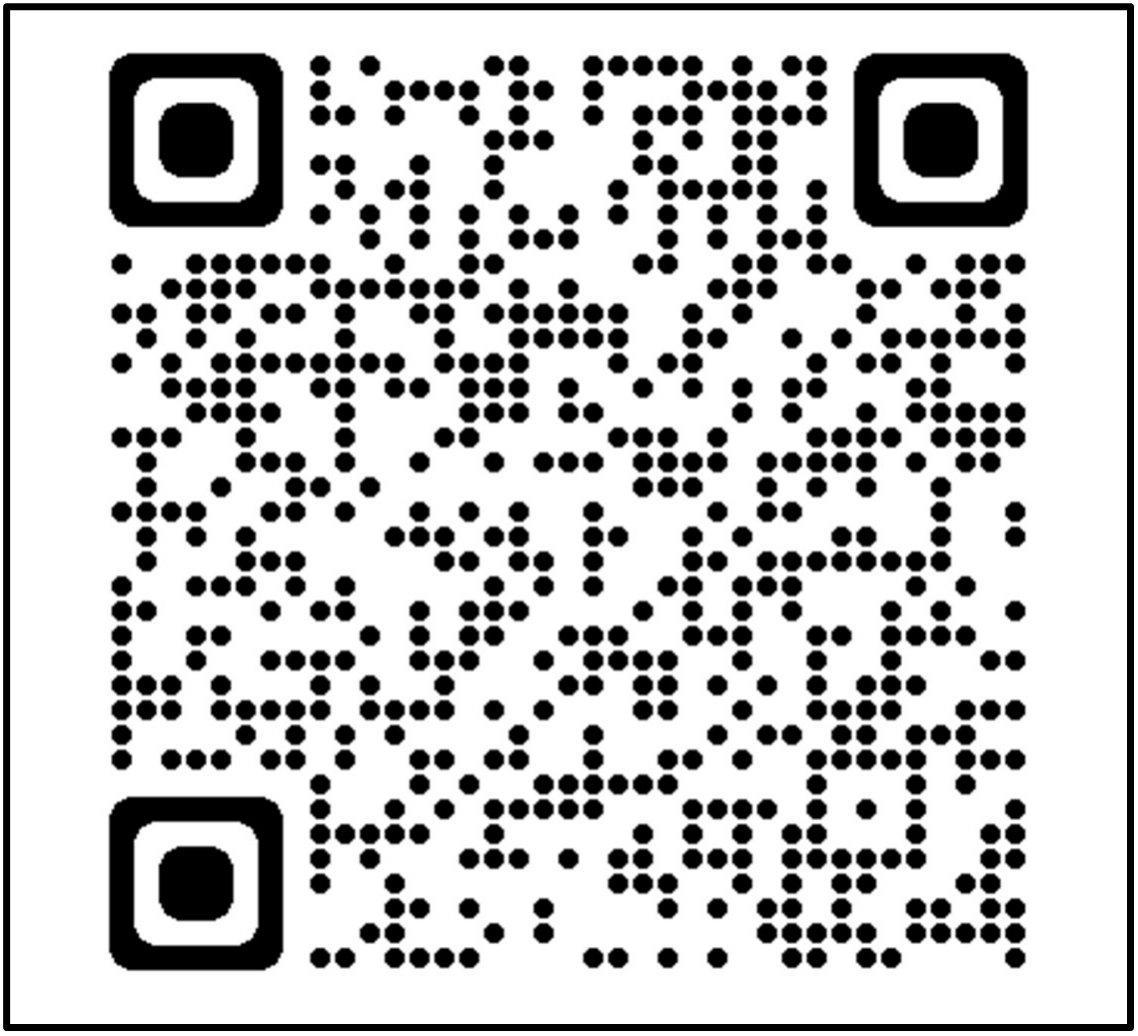
Link to app visualising the power to detect PBO synergism.

For investigating the power of a ‘4x4 tube’ bioassay design to detect a 10% difference in mortality between pyrethroid-only and pyrethroid-PBO treatments, a simulation framework was developed. To aid interpretation, an interactive visualisation is available here: https://fmechan1.shinyapps.io/who_power_designapp/, Figure 4). Investigators using the WHO tube method to assess a population for the presence of metabolic resistance mechanisms can use this tool to design well-powered experiments to reliably detect the presence or absence of statistically significant synergism.

Using the simulation-based framework, together with assumptions about variability described above, two distinct scenarios are presented here when investigating the power of a ‘4x4’ WHO tube bioassay to detect a 10% difference in mortality. Scenario (A) is the specific case of identifying a difference in mortality from 90% to 100%. Scenario (B) is a comparison without prior knowledge about the mortality of either group (where it is powered even for the ‘worst case scenario’ where either treatment or reference has mortality close to 50% thus the point of maximum uncertainty) using assumptions about variability based on the pyrethroid-resistant strain described above.

**Figure 5.**
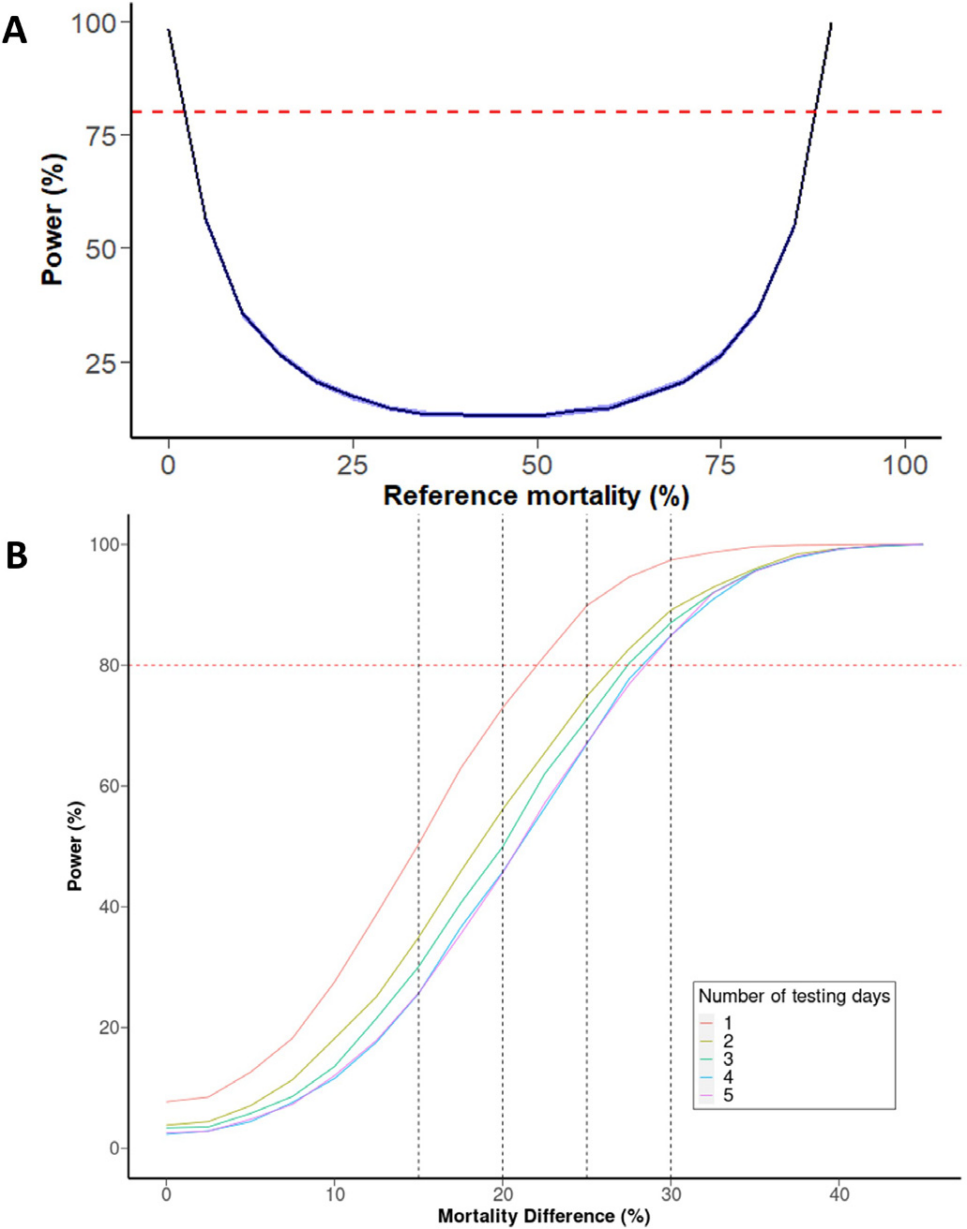
Power to detect synergism in a WHO tube assay. ( **A**) Power to detect effect sizes between PBO and non-PBO across different sample sizes. The horizontal dashed red line indicates 80% power, with vertical dashed lines indicating a 15, 20, 25, and 30% mortality difference. ( **B**) Power to detect a 10% difference in 4x4 design depending on mortality of the pyrethroid-PBO group. Blue band represents 95% CI after 10,000 iterations.

For scenario (A), when performed within a single day (within day SD= 0.25), we estimate that a 4x4 design has 96.44% chance of detecting an increase of mortality from 90% to 100% ( Figure 5A). Even if within-day SD was doubled in this comparison to 0.5 (by greater variability in mosquito size, for example), power remains sufficiently high at 95.76%.

For scenario (B), where no prior knowledge about the mortality of each treatment is known, (and thus the point of maximum uncertainty is assumed as described above) then the smallest difference that can be detected with 80% power (assuming within-day SD of 0.25 and between-day SD of 0.60) is 22.5% when performed on the same day or ~27.5% when split over multiple days ( Figure 5B). As might be expected, increasing the number of tubes of each treatment increases power however the marginal gain of each additional tube. Five tubes of each treatment ('5x5') in scenario (B) allows a 20% difference to be detected with 80% power, if performed on the same day. However, the minimum number of tubes needed to detect a 15% difference is ten of each treatment (a 10% difference required a sample size so large as to be out of the scope of our simulation framework).

As shown in Figure 5, splitting exposures over multiple days reduces power. Increasing the number of days from one to two has the largest effect as this introduces the between-day variability, but the difference between 2,3, and 4 days is small for practical purposes. Generally, splitting assays over multiple days means the mean difference must be 5% larger than otherwise to be detected with 80% power. For example, splitting a ‘5x5’ assay over multiple days increases the difference that can reliably detected from 20% to 24–26%, depending on the number of days.

## Discussion

This study aimed to quantify and identify sources of variability in WHO tube bioassays when assessing the mortality of pyrethroid-susceptible and pyrethroid-resistant *An. gambiae* exposed to permethrin and PBO. This variability information was used as the basis of a simulation framework for the sample size of tubes needed to reliably detect mortality differences. We demonstrate that the power to detect a 10% difference in mortality is dependent on what absolute mortality values are being compared (i.e. 90% vs 100% is not equivalent to 50% vs 60%). We show that a ‘4x4 design’ (four tubes of each treatment) can detect an increase in mortality from 90% to 100% with >90% power even when variance is assumed to be very high. However, this scenario where one treatment group has mean mortality close to 100% is a special edge case, as proportional data cannot have a value larger than 1 thus there is very low variance around the mean. This would also apply if mortality was close to 0%. Consequently, statistical comparisons where the mean of one group is close to 0% or 100% mortality have high power. Where both treatment groups have mortality that is not close to 0% or 100%, a 10% difference is much harder to detect. We demonstrate that for the ‘worst-case-scenario’ where one treatment group has 50% mortality (as it is furthest from 0% or 100%) the smallest mortality difference that can be detected with 80% power is 22.5%. For any middle ground where mortality for both groups is between 50% and 100%, power to detect a mortality difference can be obtained from the linked R Shiny application.

When evaluating the sources of variability, we observed that for both the pyrethroid-susceptible and resistant strain most variation occurred between testing days rather than within them. Furthermore, we observed the overall variability of the pyrethroid-susceptible Kisumu strain was greater than that of the pyrethroid-resistant Tiassalé 13 strain (when pre-exposed to PBO), with the strains having similar levels of within-day variation, but the susceptible strain approximately twice as variable between-days. However, it should be noted that due to the highly controlled rearing and testing environment, the variance estimates here are indicative of the minimum variability. Consequently, in less rigorously controlled conditions variance would be expected to be higher and thus a larger sample size needed. The R Shiny application provides sample size estimates across a range of variance assumptions.

When assessing the sources of variation in 24hr mortality, the number of mosquitoes in the tube and the total dry weight of mosquitoes were identified as predictors of the outcome, together contributing a total of approximately 10% of variability in the data. Although the WHO guidelines state that 25 mosquitoes (or as close as practically possible to 25) should be exposed in each tube, in practice this varies somewhat from tube to tube due to the challenges of manipulating live mosquitoes (which varied from 20 to 35 in our experiment). Additionally, it is recommended ( WHO, 2022f) that some measure of the size of mosquitoes used for bioassays is taken as a quality control measure and to aid the interpretation of results. Temperature and humidity, within the very narrow range of values permitted within in our climate-controlled insectary (26 ± 2 °C and 80 ± 5% RH), did not impact 24hr mortality. In conditions where temperature and humidity vary outside of these narrow ranges, temperature and humidity are well established to impact the outcome ( Mseti *et al.*, 2024). The mean wing length was not predictive of 24hr mortality in this study, with dry weight a more predictive indicator. However, in an equivalent study on WHO bottles with the same mosquito colonies run in parallel, increasing wing length was found to have a negative effect on 24-hour mortality ( Praulins *et al.*, 2024), with the same paper finding not detecting a statistical effect of dryweights on mortality. Overall, our observations indicate that approximately 90% of the variability (or ‘noise’) observed in WHO tube bioassays under these testing conditions was due to parameters not considered or quantified here. The observation that between-day variation was high, but within-day variation was comparatively low, indicates a systematic and unmeasured difference between testing days. This may be attributable to differences in the mosquitoes used on each testing day, potentially due to differences in larval environment during rearing. The number of mosquitoes in the tube was observed to have a positive effect on 24-hour mortality, which may be attributable to the greater density causing mosquitoes to collide with each other and the tube more frequently and so experience greater exposure to the insecticide ( Praulins *et al.*, 2022). Taken together, these findings indicate that performing all tests on the same day and keeping mosquito numbers consistent at 25 per tube would reduce variance by approximately two thirds, compared to allowing numbers to vary from 20 to 30 and performing the test over three days.

It was observed that 24hr mortality with the pyrethroid-resistant strain exposed to both permethrin and PBO declined with repeated use of the same permethrin-treated papers. This equated to an approximately 11% reduction with each use, up to the fourth exposure, with no subsequent decrease thereafter up to the maximum of seven. To confirm this observation this experiment was repeated in full (with the one change that papers were stored in the fridge, to remove their being stored in the first experiment with Tiassalé 13 as a potential cause), yet the decline with testing day was statistically identical across the same across the two repeats. To determine if this was due to a decline in available insecticide on the papers, chemical analysis of these samples was performed ( Table 2) which indicated no difference in total AI content on the papers across zero and seven uses, though measuring total AI content may not be sensitive enough to detect changes in surface availability which may affect bioactivity. However, it is important context that in both experiments the same cohort of mosquitoes was used for the repeated tests on the same papers, and mosquitoes tested on each day were the same age. Each subsequent test therefore used mosquitoes which had pupated on a later day than the previous test, having been reared in the same larval environment. Consequently, it is not possible to disentangle the effects of paper use with time in the larval environment (time to pupation). Future work could be done that standardised time-to-pupation to test papers multiple times, to test the hypothesis that the reduced mortality observed in this study was in fact a result of the number of times paper were used.

**Table 2.** Concentration of active ingredients in papers used for exposing Tiassalé 13.

	Mean active ingredient concentration (mg/m ^2^)(SD)							
Papers	0 uses	1 use	2 uses	3 uses	4 uses	5 uses	6 uses	7 uses
permethrin	197.33 (14.02)	312.8 (3.46)	309.27 (13.24)	312.47 (6.60)	325.58 (6.17)	331.43 (9.58)	331.78 (2.76)	332.44 (7.31)
PBO	789.7 (15.80)	758.03 (43.17)	815.63 (36.17)	738.19 (25.57)	747.11 (5.69)	732.42 (25.14)	789.04 (26.16)	781.64 (17.84)

The concern with underpowered sample sizes is not just a risk that the effect will not be detected but that comparisons that are detected (p<0.05) by underpowered sample sizes will have an inherent risk of overestimating the effect size, as has been demonstrated in previous simulation studies ( Ioannidis, 2008). Consequently, the mortality difference observed in underpowered samples is not a reliable estimate of the true effect. Current WHO guidance for identifying evidence of metabolic resistance in a mosquito population states that a 10% difference between pyrethroid-only and pyrethroid-PBO exposure in a 4x4 WHO tube assay is indicative of PBO synergism and so metabolic resistance. Where pyrethroid-only mortality is 90% and pyrethroid-PBO is 100%, our simulation study indicates that this 10% difference can be reliably detected with >90% power even when assuming a very high level of within-day variation. However, under the ‘worst-case’ scenario where the mortality of either group is 50% (assuming the level of variation observed in our assays with the pyrethroid-resistant strain), a 4x4 design is only powered to detect a 22.5% difference with 80% power. Additionally, our simulation study determined that if the exposures are performed over multiple days, rather than simultaneously within the same day, the synergistic effect must be approximately 5% larger than otherwise to be reliably detected. Finally, it should be noted that that statistical significance is not the same as biological significance, thus a difference that can not be reliably detected by the analysis may still be important in practical terms.

As this work on noise in WHO tube assays was done in parallel with an equivalent study on WHO bottle assays using the same mosquito colonies, insecticides and operators ( Praulins *et al*., 2024 *in review*), a direct comparison can be made between the variability of each bioassay. In the bottle, the overall coefficient of variation for the susceptible Kisumu strain was 0.682 compared to 0.719 observed here for the tube test, making the assays very similar in overall variability. However, the proportion of this variation that is attributable to within-day and between-day is slightly different between assays. While in both assays most variation is between days rather than within them, this ratio is particularly pronounced in the bottle assay where standard deviation within:between was 0.350:1.236 for the tube compared to 0.145:1.788 for the bottle. This may indicate that while the bottle assay itself is more consistent than the tube assay, the coating of the bottles may introduce greater variation compared to the making of insecticide papers used in tube assays.

The power simulation approach used here demonstrates a framework for taking a dataset of observations for a given bioassay and using it to produce sample size guidance for conducting an assay that is powered to detect a defined mortality difference. While typically sample size calculations are performed by individual research groups for a given planned experiment, potentially resulting in inconsistent quality and robustness, this framework allows an accessible, uniform approach. The end-user need only input their target effect size through a user interface, either accepting default assumptions about variability or manually setting these if they have specific estimates from their own lab. This ensures a uniformity of robustness and allows results to be compared across time or between studies. The underlying statistical methods can also be updated over time as guidance on how to perform the assay changes. Additionally, as all simulations have been performed in advance, the computational demands to perform the power analysis are greatly reduced for the end user, who simply needs to open a web browser to use the application. As the WHO tube bioassay is arguably the most straightforward of all WHO mosquito bioassays in terms of designing power simulations, it is ideal for a functioning proof-of-concept to adapt for other bioassay designs. The broad approach is readily applicable to other laboratory bioassays such as WHO cones or tunnel tests, and semi-field experiments such as Experimental Hut Trials ( Challenger *et al.*, 2023).

## Ethical approval and consent

Ethical approval and consent were not required.

## Data Availability

Zenodo: I2i Noise Bioassay Datasets, https://zenodo.org/doi/10.5281/zenodo.12772204, ( Mechan & Praulins, 2024) This project contains the following underlying data: Noise_Bottle_AllTimepoints.csv Noise_Tube_Kisumu.csv Noise_Tube_Tiassale.csv Data are available under the terms of the Creative Commons Attribution 4.0 International license (CC-BY 4.0)( https://creativecommons.org/licenses/by/4.0/).

## Software availability

Source code available from: https://github.com/i2i-Data-Repository/WHO-Tube-Bioassay

Archived source code is available from https://doi.org/10.5281/zenodo.12700083 ( Mechan, 2024)

Source code is available under the terms of the GNU General Public License version 3 (GPL-3.0-only)( https://opensource.org/license/gpl-3-0).
