# Supplementary material for: Power calculation for mosquito bioassays: Quantifying variability in the WHO tube bioassay and developing sample size guidance for the PBO synergism assay using a Shiny application
Source: Gates Open Res. 2024 Sep 12;8:96. [Version 1] doi: 10.12688/gatesopenres.16123.1 (PMC11576473; doi:10.12688/gatesopenres.16123.1)
Supplement: Peer review history

# Reviewer report: Reviewer response for version 1

**DOI:** 10.21956/gatesopenres.17486.r38354

**Published:** 2024-11-25

**Authors:** Jirod Nararak

**Affiliations:** 1 Entomology, Kasetsart University, Bangkok, Thailand

The research article presents a detailed analysis of variability in the WHO tube bioassay for measuring insecticide resistance in mosquitoes and establishes guidance on sample sizes necessary for detecting synergistic effects with the PBO synergism assay. Using data from *Anopheles gambiae* strains (both pyrethroid-susceptible and resistant), the study identifies that most variability occurs between testing days rather than within them, and that factors like mosquito number and body weight contribute minimally to this variability. A simulation-based power analysis framework is developed, demonstrating that the traditional “4x4” tube design is sufficient to detect a 10% increase in mortality when comparing groups with mortality near 100%.

 However, for more challenging scenarios—such as distinguishing a 10% difference at mid-range mortality (e.g., 50%)—a larger effect size is required for reliable detection.

 The study provides a Shiny application for users to calculate optimal sample sizes for various scenarios, allowing better-informed decisions on PBO-treated ITNs’ deployment in resistance management. The findings suggest that, while the current WHO guidelines are adequate in ideal cases, they may require revision in scenarios with higher baseline variability or when distinguishing smaller effect sizes.

Reviewer Decision:

 Given its comprehensive analysis, innovative approach in providing a user-friendly tool for sample size calculations, and the relevance of its findings to global malaria vector control strategies, this article is well-suited for indexing. It offers valuable contributions to the field by refining methodologies in resistance monitoring and could inform updates to WHO guidelines for bioassays.

Is the work clearly and accurately presented and does it cite the current literature?

Yes

If applicable, is the statistical analysis and its interpretation appropriate?

Yes

Are all the source data underlying the results available to ensure full reproducibility?

Yes

Is the study design appropriate and is the work technically sound?

Yes

Are the conclusions drawn adequately supported by the results?

Yes

Are sufficient details of methods and analysis provided to allow replication by others?

Yes

Reviewer Expertise:

Mosquito behavior, Mosquito ecology, Mosquito repellent

I confirm that I have read this submission and believe that I have an appropriate level of expertise to confirm that it is of an acceptable scientific standard.

# Reviewer report: Reviewer response for version 1

**DOI:** 10.21956/gatesopenres.17486.r38348

**Published:** 2024-11-19

**Authors:** Olukayode G Odufuwa

**Affiliations:** 1 Vector Biology, Swiss Tropical and Public Health Institute, Basel, Switzerland

*Page refers to the page number where the statement that requires revision is located in the document attached*

*Section: At the stage of paper review, other journals usually request authors to use line numbers in their manuscript so that it is easy to direct authors to where the statement in question should be addressed. In this case, there is none. So I used sections to mean where the statement can be found: abstract, introduction, method, result or discussion.*

*Text refers to the authors' statement in the manuscript that should be revised or in question.*

*Comment: refers to my statement as a reviewer.*

**Review of Power calculation for mosquito bioassays: Quantifying variability in the WHO tube bioassay and developing sample size guidance for the PBO synergism assay using a Shiny application**

 The study investigated the sources of variability in tube bioassay, a method used in detecting mosquito susceptibility to concentrations of pyrethroid insecticides or piperonyl butoxide (PBO) synergist. The results are important knowledge for the community of vector control experts to allow critical thinking of study variability when designing tube bioassay studies and the R shiny application is a very useful tool as many labs lack this expertise and often conduct studies without considering study power.

 I recommend the paper for publication with minor changes.       

 Below are my comments. Revision was done using the PDF version. Note that the manuscript didn’t have lines number, so it is not straightforward to indicate the text that requires comments. If possible, next submission should  have it so that reviewers will be able to easily direct authors to the location of the texts in question.

1. **Major compulsory revisions**

Page

 Section

 Text

 Comment

 4

 WHO tube bioassay

 The permethrin concentration for the pyrethroid-resistant

 Tiassalé 13 strain was the WHO discriminating concentration

 of 0.05% (WHO, 2022e)

 The discriminating concentration suggested in the referenced guidelines for permethrin is 0.75% and not 0.05%. Authors need to provide another justification for the selected concentration.

 Also, 0.75% should also be considered for susceptible mosquitoes in addition to the already selected concentration of 0.03% to reflect reality. Given that few labs are already complaining of being able to rear 100 mosquitoes for these tests, this will help to indicate that rigorous power is only needed for resistant strain and not susceptible if all mosquitoes are dying.

 2) **Minor compulsory revisions**

 Page

 Section

 Text

 Comment

 3

 Introduction

 Paragraph 1

 Efficacy

 Authors may attempt to add a simple definition of efficacy in bracket to help non-experts readers understand quicker.

 4

 WHO tube bioassays

 Each tube was scored for knockdown at 60 minutes

 Comment on when sugar was offered to the mosquitoes after one hour exposure in the tubes and assessment of knockdown. Given that the time sugar is offered to these mosquitoes may influence their responses to insecticide.

 4

 Statistical analysis

 The explanatory variables assessed

 in the GLMM

 Authors may comment on if the same technician conducted the tests throughout the period of testing. This does not have to be included in the paper. However, if different technicians conducted the test, the variability contributed by technicians is worth investigating.

 5

 results

 (was not assessed for Kisumu)

 Authors may add a sentence on why it was not assessed for Kisumu.

 8

 Discussion

 the overall variability of the

 pyrethroid-susceptible Kisumu strain was greater than that of

 the pyrethroid-resistant

 Authors may add to the discussion section if the differences in the concentrations contributed to the overall variability difference between the strains observed in the study given that concentration was different.

 5

 Discussion

 Authors may discuss the reason for the lower proportion of mortality for susceptible mosquitoes ( 49%) compared to the resistant (53%). Is this due to the different concentration?

 8

 Discussion

 Authors should comment in the discussion section if findings is applicable to other types of pyrethroids like deltamethrin and alpha cypermethrin, given that these insecticides are more likely to induce higher mosquito mortality than permethrin.

 3) **Minor revisions**

 Page

 Section

 Text

 Comment

 1

 Abstract background

 WHO

 Write in full World Health Organization

 1

 Abstract background

 mortality

 Change to “mosquito mortality”

 Authors could change mortality to mosquito throughout the papers in clauses where no mosquito is mentioned before mortality.

 2

 Abstract result

 Humidity

 Change to “relative humidity”

 Authors could use this term consistently in the paper to distinguish it from other types of humidity.

 2

 Abstract result

 didn’t impact mortality

 Change to “influence mosquito mortality”

 2

 Abstract conclusions

 assay

 Change to “bioassay”

 This term could be consistent throughout the paper.

 3

 Mosquito rearing

 environmentally controlled

 Authors have used climate-control in the abstract which means the same thing with environmentally controlled. Authors should choose one and consistently use the chosen phrase throughout the paper.

 4

 WHO tube bioassays

 though only 24hr mortality

 will be assessed here

 The text should be changed to past tense “24hr mortality was assessed here”

 5

 Statistical analysis

 A p value was

 More than one p-values were estimated in this study, so I suggest that authors could change the text to “P-values were”

 5

 results

 (tffhis was not assessed for Kisumu)

 Typo should be corrected

Is the work clearly and accurately presented and does it cite the current literature?

Yes

If applicable, is the statistical analysis and its interpretation appropriate?

Yes

Are all the source data underlying the results available to ensure full reproducibility?

Yes

Is the study design appropriate and is the work technically sound?

Yes

Are the conclusions drawn adequately supported by the results?

Yes

Are sufficient details of methods and analysis provided to allow replication by others?

Yes

Reviewer Expertise:

Vector control products' data generator.

I confirm that I have read this submission and believe that I have an appropriate level of expertise to confirm that it is of an acceptable scientific standard, however I have significant reservations, as outlined above.

# Reviewer report: Reviewer response for version 1

**DOI:** 10.21956/gatesopenres.17486.r38352

**Published:** 2024-11-18

**Authors:** Shüné V Oliver

**Affiliations:** 1 University of the Witwatersrand, Johannesburg, South Africa

The authors offer an important study with significant epidemiological consequences. PBO exposure is an important mechanism of implicating P450s in metabolic resistance, particularly in resource-limited settings. Therefore, understanding sources of variability in these assays is critical. Furthermore, the identification of the sources variability within tube assays is useful as well. In general, the document is well written and easy enough to follow. I have made a few annotations on the manuscript, but the two most significant comments I have are as follows:

 Figures 1 and 2 are do not have enough of a legend to be standalone images. There needs to be more of a descriptive legend, particularly as the two images are related and the axes are different.

 It would be useful to include in your discussion the implications of how to deal with this variability in F1 populations which would be analysed for PBO synergism. There would be less of an opportunity to control for time to pupation to reduce variability. What suggestions would you have for researchers testing local populations where this would not be possible to control.

 There are points that are made directly on the MS and this is critical to my review: https://gatesopenresearch.s3.eu-west-1.amazonaws.com/linked/218108.Id-16123-Reviewer_File.pdf

Is the work clearly and accurately presented and does it cite the current literature?

Yes

If applicable, is the statistical analysis and its interpretation appropriate?

Yes

Are all the source data underlying the results available to ensure full reproducibility?

Yes

Is the study design appropriate and is the work technically sound?

Yes

Are the conclusions drawn adequately supported by the results?

Yes

Are sufficient details of methods and analysis provided to allow replication by others?

Yes

Reviewer Expertise:

Vector biology, Insecticide resistance

I confirm that I have read this submission and believe that I have an appropriate level of expertise to confirm that it is of an acceptable scientific standard.

# Reviewer report: Reviewer response for version 1

**DOI:** 10.21956/gatesopenres.17486.r38132

**Published:** 2024-10-25

**Authors:** Leslie C Rault

**Affiliations:** 1 University of Nebraska-Lincoln, Lincoln, USA

This study focuses on detecting some of the factors causing variability and reducing power in analysis of mortality and synergistic assays when using the WHO tube assay. The goal is to give guidance on how to generate robust data for resistance detection and PBO synergy to detect metabolic resistance particularly.

 I believe that the manuscript is well written, with good grammar, and is straightforward. I would not pretend to be a statistician, so I would leave analysis of some of the method to someone more qualified but I do have experience with insect bioassays, particularly mosquitoes. Thus, I do have some comments pertaining to my areas of expertise and just general questions/concerns.

 1. In the introduction:

 When mentioning PBO as the way to go to detect resistance that "results from upregulation of P450 metabolic enzymes" I don't necessarily agree because PBO also has some effect on esterases so I would just rephrase "results in increased activity of metabolic enzymes, i.e. P450s and esterases".

 2. In the methods:

 Citing Williams et al for rearing lacks some precision on what blood was used for rearing the colonies used in this study.

 Latin words should always be italicized: " *ad libitum*"

 When referring to the general assay used here, it seems to follow exactly the WHO guidelines, however, the concentration used for the two strains was different and was their respective LC _50_, not the 0.75% DC recommended for permethrin. So make this clear. Similarly, did you also use the 1h holding tube first stage of the assay? Please address.

 When describing the dry weight scoring, could you explain better the set up with silica gel and how this is relevant to collecting the weights?

 Additionally, you mention that the synergism assay was simulation based? Then how does is come back in the rest of the manuscript like an assay you did run in tube assay? Please make it clear.

 3. Results:

 When you talk about the differences between the two repeats and how paper was either stored in the room or in the fridge, could it also be an explanation for some of the differences? And also in the first instance it is mentioned, make sure it is clear you are talking about two repeats on different days for the number of uses of the paper (may need to rephrase this).

 4. Discussion

 Clear and straightforward, except when dealing with the PBO exposure assays, it sounds like you did run the assay, if it is the case, it should be clear in methods and in results.

Is the work clearly and accurately presented and does it cite the current literature?

Yes

If applicable, is the statistical analysis and its interpretation appropriate?

I cannot comment. A qualified statistician is required.

Are all the source data underlying the results available to ensure full reproducibility?

No source data required

Is the study design appropriate and is the work technically sound?

Yes

Are the conclusions drawn adequately supported by the results?

Yes

Are sufficient details of methods and analysis provided to allow replication by others?

Partly

Reviewer Expertise:

general entomology, toxicology, insect physiology and biology

I confirm that I have read this submission and believe that I have an appropriate level of expertise to confirm that it is of an acceptable scientific standard, however I have significant reservations, as outlined above.
